# The Impairment of Methyl Metabolism From *luxS* Mutation of *Streptococcus mutans*

**DOI:** 10.3389/fmicb.2018.00404

**Published:** 2018-03-12

**Authors:** Xuchen Hu, Yuxia Wang, Li Gao, Wenxin Jiang, Wenzhen Lin, Chenguang Niu, Keyong Yuan, Rui Ma, Zhengwei Huang

**Affiliations:** ^1^Department of Endodontics, Ninth People's Hospital, Shanghai Jiao Tong University School of Medicine, Shanghai Key Laboratory of Stomatology & Shanghai Research Institute of Stomatology, National Clinical Research Center of Stomatology, Shanghai, China; ^2^Department of Endodontics, Tianjin Stomatological Hospital, Nankai University, Tianjin, China

**Keywords:** *Streptococcus mutans*, methionine metabolism, LuxS, SahH, high-performance liquid chromatography–tandem mass spectrometry

## Abstract

The *luxS* gene is present in a wide range of bacteria and is involved in many cellular processes. LuxS mutation can cause autoinducer(AI)-2 deficiency and methyl metabolism disorder. The objective of this study was to demonstrate that, in addition to AI-2-mediated quorum sensing (QS), methyl metabolism plays an important role in LuxS regulation in *Streptococcus mutans*. The *sahH* gene from *Pseudomonas aeruginosa* was amplified and introduced into the *S. mutans luxS*-null strain to complement the methyl metabolism disruption in a defective QS phenotype. The intracellular activated methyl cycle (AMC) metabolites [S-adenosylmethionine (SAM), S-adenosylhomocysteine (SAH), homocysteine (HCY), and methionine] were quantified in wild-type *S. mutans* and its three derivatives to determine the metabolic effects of disrupting the AMC. Biofilm mass and structure, acid tolerance, acid production, exopolysaccharide synthesis of multispecies biofilms and the transcriptional level of related genes were determined. The results indicated that SAH and SAM were relatively higher in *S. mutans luxS*-null strain and *S. mutans luxS* null strain with plasmid pIB169 when cultured overnight, and HCY was significantly higher in *S. mutans* UA159. Consistent with the transcriptional profile, *luxS* deletion-mediated impairment of biofilm formation and acid tolerance was restored to wild-type levels using transgenic SahH. These results also suggest that methionine methyl metabolism contributes to LuxS regulation in *S. mutans* to a significant degree.

## Introduction

**Figure 1:**
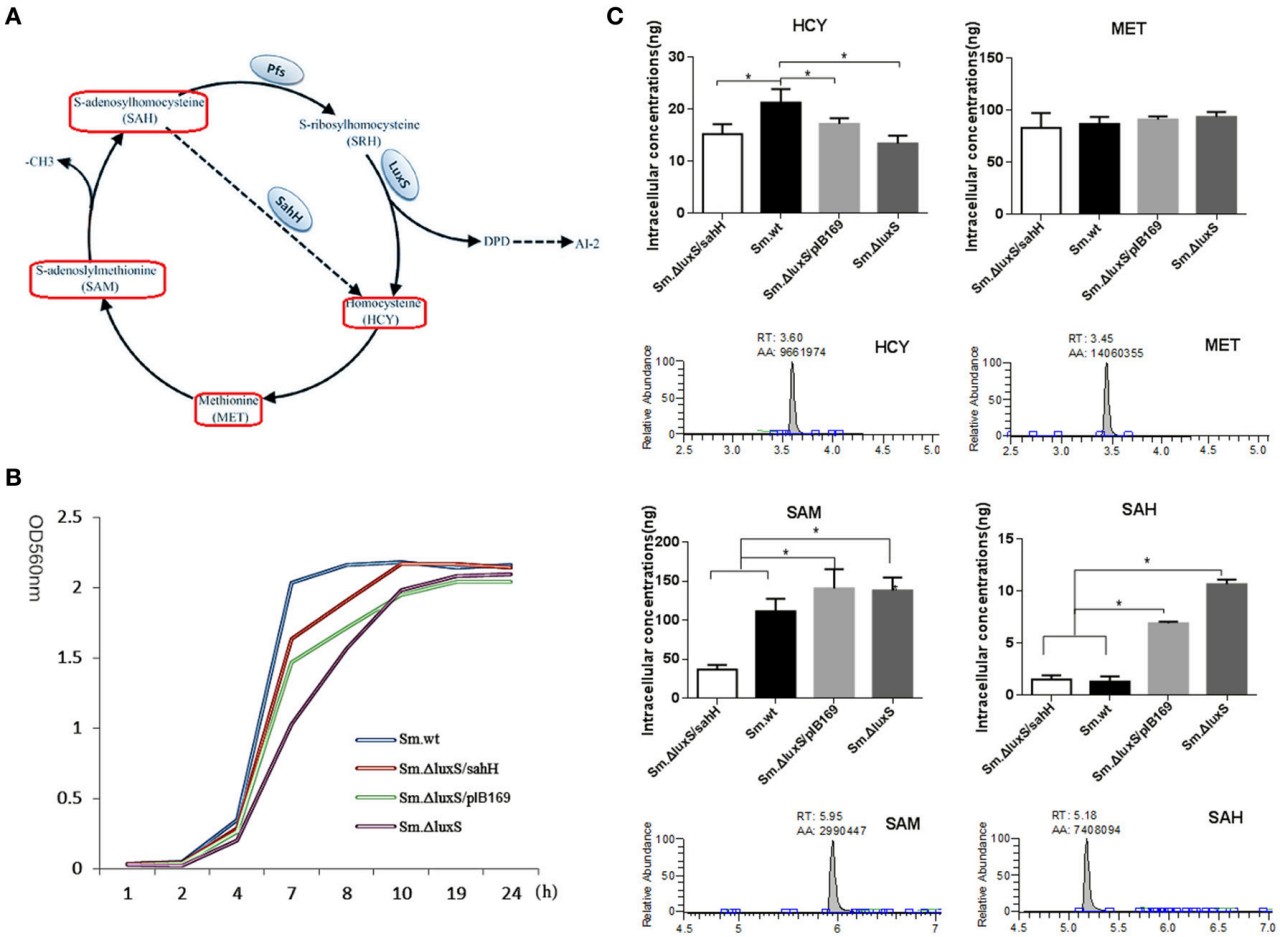
Intracellular concentration of four activated methyl cycle (AMC)-related metabolites in wild-type *Sm*.wt and derivative strains. **(A)** The AMC. The detected compounds are circled on the diagram (red box). **(B)** Bacterial growth curve of the four strains. **(C)** Intracellular concentrations of the four metabolites and their representative chromatograms. Cell extracts were analyzed with HPLC-MS/MS to determine the intracellular concentrations of methionine, homocysteine, S-adenosyl methionine, and S-adenosyl homocysteine when bacterial strains are cultured overnight and compared to their chromatographic standards. The data shown correspond to the mean calculated intracellular concentrations ± standard deviations of three independent cultures. ^*^Significant differences at *P* < 0.05.

*Streptococcus mutans* is naturally present in the human oral microbiota and is considered a primary etiological agent of caries, which is the most prevalent oral disease (Loesche, 1986; Ghasempour et al., 2013). The coordination of communication and group behavior in *S. mutans* has a significant impact on the cariogenic ability of this bacterial species (Li et al., 2008). Quorum sensing (QS) is a well-known cell-to-cell communication mechanism defined as a process whereby cells collectively regulate gene expression by producing, secreting, and responding to the accumulation of small chemical signal molecules known as autoinducers (AIs) (Bassler, 1999). Among the potential QS signal molecules, AI-2 is produced by a broad range of bacteria and may participate in interspecies communication (Blehert et al., 2003; Merritt et al., 2003). AI-2 is a byproduct of the activated methyl cycle (AMC; Figure 1A), which generates activated methyl groups for the methylation of DNA, RNA, and proteins (Winzer et al., 2002; Sun et al., 2004; Parveen and Cornell, 2011; Redanz et al., 2012). In the AMC, S-adenosylmethionine (SAM), the main methyl donor, is catalyzed by the enzyme MetK to form S-adenosine homocysteine (SAH). Then SAH is converted into homocysteine (HCY) through a two-step process. SAH uses the enzyme Pfs to generate S-ribosyl-homocysteine, then converted into homocysteine (HCY) by the enzyme LuxS (Díaz et al., 2014). For bacteria without LuxS/Pfs pathway, including *Pseudomonas aeruginosa*, S-adenosyl-L-homocysteine hydrolase (SahH) encoded by *sahH* is used to complete the AMC (Cataldi et al., 2009; Fernandez-Sanchez et al., 2009). In contrast to the LuxS/Pfs-dependent two-step pathway, SahH directly catalyzes the toxic intermediate SAH into HCY without generating AI-2 (Wang et al., 2012).

By knocking out *luxS* from the genome, many studies have shown that LuxS mutation impairs biofilm formation, acid tolerance, and acid production, and this impairment is the result of AI-2 mediated QS (Wen and Burne, 2004; Yoshida et al., 2005; Huang et al., 2009). However, in the AMC, deletion of *luxS* may cause metabolic disruption and AI-2 deficiency. Therefore, a debate about whether changes caused by *luxS* mutation should be attributed solely to QS disruption has arisen. Yoshida et al. (2005) observed that the LuxS defect could be rescued by using supernatants from *Streptococcus gordoniae* and *Streptococcus sorbrinus*, which produce AI-2. Similar results were obtained by Rickard et al. (2006) by using synthetic AI-2, suggesting a role of QS in the alterations. However, some studies reported a lack of effect of synthetic AI-2 on the changes induced by LuxS mutation. For example, in a study of *S. mutans*, 30% of the genes altered by *luxS* mutation, including genes related to biofilm formation and acid tolerance, were not restored by synthetic AI-2 supplementation (Sztajer et al., 2008). Similar results were observed in *Salmonella enterica* (De Keersmaecker et al., 2005) and *Lactobacillus rhamnosus* (Lebeer et al., 2007). Moreover, a genomic analysis by Rezzonico (Rezzonico and Duffy, 2008) revealed that the AI-2 receptor was restricted to *Vibrionales*, suggesting that *luxS* has a role other than QS in most bacteria.

Based on the contradictory evidence of the predominant role of QS in *luxS* mutation-induced changes and the evidence suggesting the absence of AI-2 receptors in most bacterial species (Blehert et al., 2003), we hypothesized that metabolism plays a critical role in LuxS-based changes in *S. mutans*. Since *S. mutans* does not have a *sahH* gene, to confirm this hypothesis, the exogenous *sahH* gene from *P. aeruginosa* was used to complete the disrupted AMC in an *S. mutans luxS*-null strain, and consequently to distinguish the contribution of metabolism from the original functions of LuxS. The hypothesis was verified by determining whether metabolism, phenotypes, and gene expression were restored. To the best of our knowledge, this experimental approach has not been used previously.

## Materials and methods

### Bacterial strains and growth conditions

The bacterial strains and plasmids used in this study are listed in Table 1. *Escherichia coli* and *P. aeruginosa* were grown in Luria-Bertani (LB) medium, and chloramphenicol (20 μg/mL) was added when necessary. *S. mutans* UA159 (wild-type; *Sm*.wt) and its derivatives were routinely grown in Brain Heart Infusion (BHI) medium (Oxoid, Wesel, Germany) with or without erythromycin 10 μg/mL or chloramphenicol 20 μg/mL. For transformation, a *S. mutans luxS*-null strain (*Sm*.Δ*luxS*) was cultured in Todd-Hewitt broth (Becton, Dickinson and Company) with yeast extract (0.2% w/v), heat-inactivated horse serum (5% v/v), and erythromycin (10 μg/mL). The transformation products were grown on Mitis Salivarius (MS) agar (Becton, Dickinson and Company) with erythromycin (10 μg/mL), and chloramphenicol (20 μg/mL). For biofilm assay, semi-defined biofilm medium (BM) containing glucose (0.8% w/v), and sucrose (0.5% w/v) (BMGS) as the alternative carbohydrate source was used (Wen and Burne, 2004). The primers used in this study are listed in Table 2.

**Table 1:** Strains and plasmids used in this study.

**Strains and plasmids**	**Relevant characteristics or distributions**	**Source or reference**
**STRAINS**		
***Streptococcus mutans***		
*Sm*.wt	*Streptococcus mutans* UA159 wide type	Huang et al., 2009
*Sm*.Δ*luxS*	*luxS::erm*AM	This study
*Sm*.Δ*luxS*/*sahH*	*luxS::erm*AM; pIB169Cm^r^-*sahH*	This study
*Sm*.Δ*luxS*/pIB169	*luxS::erm*AM; pIB169*Cm^*r*^*	This study
***Pseudomonas aeruginosa***		
PAO1	Wide type	Cataldi et al., 2009
***Escherichia coli***		
TOP10	Cloning host	Wang et al., 2012
**PLASMIDS**		
pIB169	*E. coli*-streptococcal shuttle vector, Cm^r^	Biswas et al., 2008
pIB-*sahH*	pIB169 carrying *sahH*, Cm^r^	This study

**Table 2:** Primers used in this study.

**Primers**	**Nucleotide sequence**	**Gene, description, and product size**
SF^*^	5′-CCG**GAATTC**ATGAGCGCTGTCATGACG-3′	*sahH* gene; 1427 bp
SR^#^	5′-CGC*GGATCC*TTAGTAGCGATAGGTGTCCGG-3′	
*16S*-F	5′-CACACCGCCCGTCACACC-3′	16S rRNA,
*16S*-R	5′-CAGCCGCACCTTCCGATACG-3′	normalizing internal standard. 160 bp
*smu.44*-F	5′-ATTGGTAGATTATCACTTGGCAGAC-3′	*smu.44*,
*smu.44*-R	5′-AGGCAAACTCACTCATTGACAAC-3′	DNA mismatch repair protein. 166 bp
*smu.46*-F	5′-GAGGTATTCACCAAGGAAGATG-3′	*smu.46*,
*smu.46*-R	5′-TCCGTCGAAAAAGCATCAGACT-3′	LuxR-type regulator. 137 bp
*ciaH*-F	5′-CGTCATCAATAATGTCAATGCCTTC-3′	*ciaH*,
*ciaH*-R	5′-TACCTTAACTGTCACTGTCCGATAC-3′	histidine kinase sensor. 139 bp
*aguA*-F	5′-AAGGTTTGTGAAATAGAAGGTGTGG-3′	*aguA*,
*aguA*-R	5′-CTTTGGTCAGATGCGGATTACG-3′	agmatine deiminase. 148 bp
*smu.238*-F	5′-TTTGATGGGCGTGAAGCATTAAG-3′	*smu.238*,
*smu.238*-R	5′-AGCACCAATTTCAAGACCAATAACC-3′	membrance transport. 19 2bp
*gtfB*-F	5′-TGCCGCAGTCCCTTCTTATTC-3′	*gtfB*,
*gtfB*-R	5′-GCCATGTATTGCCCGTCATCT-3′	glucosyltransferase. 287 bp
*gtfC*-F	5′-GTGCGCTACACCAATGACAGAG-3′	*gtfC*,
*gtfC*-R	5′-GCCTACTGGAACCCAAACACCTA-3′	glucosyltransferase. 108 bp
*gtfD*-F	5′-TGGCACCGCAATATGTCTCTTC-3′	*gtfD*,
*gtfD*-R	5′-CAATCCGCAATAACCTGAATACCG-3′	glucosyltransferase. 184 bp
*gbpA*-F	5′-TACAGTTGAGGCTCGTTTCCC-3′	*gbpA*,
*gbpA*-R	5′-CCGTCATCAGGCACAGAACC-3′	glucan binding protein. 171 bp
*gbpC*-F	5′-ACACCACCAACAACTCCTGATG-3′	*gbpC*,
*gbpC*-R	5′-CACGCTCTCTAACACGCATTTC-3′	glucan binding protein. 133 bp
*ldH*-F	5′-ACTTCACTTGATACTGCTCGTT-3′	*ldH*,
*ldH*-R	5′-AACACCAGCTACATTGGCATGA-3′	lactate dehydrogenase. 123 bp

* *EcoRI restriction sites are in boldface*.

# *BamHI restriction sites are underlined*.

### Construction of the cloning vector and growth curve

*sahH* were amplified from the genomic DNA of *P. aeruginosa* PAO1. The *E. coli*-streptococcal shuttle plasmid pIB169 was kindly provided by Indranil Biwas (University of Kansas Medical Center, Kansas, USA) (Biswas et al., 2008). After DNA digestion and ligation, *sahH* were cloned into pIB169, generating the plasmids pIB-*sahH*. After sequencing, pIB-*sahH* and pIB169 were individually transformed into *Sm*.Δ*luxS* using the competence-stimulating peptide (CSP), and generating two *S. mutans*-derived strains: *Sm*.Δ*luxS*/*sahH* (*S. mutans luxS*-null strain with pIB-*sahH*) and *Sm*.Δ*luxS*/pIB169 (*S. mutans luxS*-null strain with pIB169). CSP (amino acid sequence: SGSLSTFFRLFNRSFTQALGK) (Cvitkovitch, 2001) was synthesized by GL Biochem (Shanghai, China). The protocol for CSP transformation was described previously (Biswas et al., 2007). The transformants were selected on MS agar using erythromycin (10 μg/mL) and chloramphenicol (20 μg/mL). The growth curves of these four strains were determined in BHI medium under anaerobic conditions.

### Extraction of metabolites

Ice-cold isotonic saline was used to wash the *S. mutans* cultures twice to remove the extracellular components present in the growth medium. After that, 1 mL of an ice-cold extracting solution (methyl alcohol:acetonitrile:water ratio of 4:4:2 v/v/v, containing 0.1% methane acid) and 0.7 g of acid-washed glass beads were mixed with the bacterial cells for the mechanical rupture of the cell walls. Moreover, an ice-cold extracting solution was used to quench and permeabilize the cells, inhibit any residual enzymatic activity by protein precipitation, and maximize the extraction of AMC metabolites. The mixture was centrifuged at 12,000 *g* for 20 min at 4°C, and the supernatant was collected. The procedure was repeated twice, and all the supernatants were pooled. Subsequently, the supernatants were treated by adding 20 μL of 1 mol/L dithiothretiol and incubated at 65°C for 30 min to ensure the complete reduction of homocysteine (Jiang et al., 2017). The obtained solution was freeze-dried and stored at −80°C. A spectrophotometric method for the determination of tryptophan (Edelhoch, 1967) was used to quantify the residual protein of bacterial sediments, maintain an identical biomass between individual samples, and improve the batch-to-batch consistency of the method.

### HPLC-MS/MS analysis

Stock solutions of SAM, SAH, HCY, methionine (MET), and [^13^CD_3_] methionine ([^13^CD_3_]MET) ([^13^CD_3_]MET is not a hazardous substance or mixture according to Regulation (EC) No. 1272/2008) (Sigma-Aldrich, St. Louis, MO, USA) were prepared at 1 mg/mL in water. A standard solution was prepared by combining the stock solutions and diluting them with water immediately before use. The working standard solutions were 2, 10, 20, 50, 500, and 1,000 ng/mL. [^13^CD_3_]MET at a concentration of 200 ng/mL in water was used as the internal standard (IS) (Halliday et al., 2010). All stock solutions were stored at −80°C. Before analysis, the samples were redissolved in 1 mL of water. The samples and standards were centrifuged at 12,000 *g* for 5 min at 4°C. A 30-μL aliquot of each supernatant was transferred to HPLC vials. A triple quadrupole Quattro Ultima mass spectrometer (Thermo Fisher Scientific, Waltham, MA, USA) and an ACQUITY UPLC BEH Amide Column (2.1 × 100 mm, 1.7 μm) (Waters, Milford, USA) were used to quantify the intracellular AMC metabolites. The column was maintained at 30°C and the mobile phase flow rate was set at 200 μL/min. The gradient elution mobile phases were methyl alcohol (solution A) and 0.1% methane acid in acetonitrile (solution B). The following ramped gradient was used: 0 min at 95% A; 0.5 min at 95% A; 8 min at 5% A; 10 min at 5% A; 11 min at 95% A; and 13 min at 95% A (Da Silva et al., 2016). The analytes were quantified using tandem electrospray mass spectrometry in the positive mode (+ESI). The pressure of the collision gas (argon) was set at 1.5 mTorr. The software Xcalibur version 2.1 was used for instrument control, data acquisition, and data analysis.

### Quantification of biofilm formation

Crystal violet (CV)-staining was used to quantify biofilm formation by *S. mutans* (7, 35). *Sm*.wt and its derivatives were grown in BHI broth overnight at 37°C under anaerobic conditions. After initial dilutions to achieve a similar cell density (OD_560_ = 0.8), the cultures were diluted 1:10 with fresh BMGS medium. Two-hundred microliters of the cell dilutions were transferred to the 96-well flat-bottom microtiter plate (Corning, Corning, NY, USA). Wells containing uninoculated growth medium were used as negative controls. The plates were incubated at 37°C under anaerobic conditions for 16–24 h without agitation. The liquid medium was removed and the wells were gently rinsed three times with sterile distilled water to remove the planktonic or loosely bound cells. The plates were air-dried and stained with 0.1% (w/v) CV (50 μl per well) for 15 min at room temperature. Excess dye was removed and the plates were rinsed three times with distilled water. After air-drying, 200 μl of 99% ethanol was added to each experimental well and the plates were shaken for several minutes to induce dye release. The amount of biofilm was determined by spectrophotometric reading at OD_570_ using a Varioskan Flash plate reader (Thermo Fisher Scientific). Each assay was performed in triplicate.

### Analysis of biofilm by confocal laser scanning microscopy

Culture dilutions were prepared using the quantitative assay protocol described above. Alexa Fluor 647-labeled dextran conjugate (Ren et al., 2016) (1 M; Life Technologies, Carlsbad, CA, USA) was added to the BHIS medium before inoculation. One milliliter of each diluted culture was transferred to individual wells of a 24-well flat-bottom plate with a glass coverslip on the bottom of each well. The plate was incubated at 37°C overnight under aerobic conditions. The biofilm was stained with a MSYTO 9 green fluorescent nucleic acid stain (Life Technologies). After incubation at room temperature in the dark for 15 min, all samples were scanned using a Leica TCS SP2 confocal laser scanning microscope (CLSM, Leica, Wetzlar, Germany) at 1-μm intervals, and images were recorded from the moment the signal appeared until the signal disappeared. The images were randomly captured from each sample.

### Quantitation of extracellular polysaccharides

*Sm*.wt and its derivatives were cultured under anaerobic conditions in 24-well flat-bottom culture plates overnight at 37°C. After incubation, the culture medium was carefully removed and replaced with 2 mL of sterile PBS. The cells were resuspended and vortexed. The suspension was centrifuged at 6,000 g for 10 min at 4°C and the supernatant was collected. This procedure was repeated twice and all the supernatants containing the water-soluble extracellular polymeric substances (EPS) were pooled. Water-insoluble EPS was extracted from the samples using 1.0 M NaOH with agitation for 2 h at 37°C, as previously described (Ren et al., 2016). The concentration of alkali-soluble carbohydrate in the supernatant was determined using the anthrone-sulfuric method (Chen et al., 2017). Cell dry weight was measured to standardize the number of bacteria.

### Assessment of acid tolerance

Acid tolerance was assessed by exposing the biofilms to an acidic environment. Biofilms were incubated in 96-well plates as described for the quantitative biofilm assay. After incubation at 37°C under aerobic conditions without agitation for 8 h, the supernatants were removed and the formed biofilm was rinsed with sterile double distilled water. Fresh BMGS at pH 5.8, 4.3, and 2.8 in series was added to the wells, and the plates were incubated for 24 h. BMGS medium (pH 7.4) was used as a standard control. The resultant biofilm was quantified using the quantitative assay protocol. The decrease in biofilm formation at lower pH-values (5.8, 4.3, and 2.8) compared with the respective neutral controls was calculated to determine the acid suppression effect.

### Measurement of lactic acid production

The disks containing biofilms were rinsed with cysteine peptone water to remove loose bacteria. Each disk was placed in a new 24-well plate with 1.5 mL of buffered peptone water (BPW) supplemented with 0.2% sucrose. BPW medium was used to maintain the stability of the biofilms during the 3-h incubation. BPW is suitable for this purpose because its relatively high buffering capacity prevents the decrease in pH, and low pH hinders bacterial acid production. Disks with biofilms were incubated at 37°C for 3 h to allow the biofilms to produce acid. After 3 h, the BPW solutions were stored for later lactate analysis using an enzymatic (lactate dehydrogenase) method (Powers et al., 2007). A microplate reader (SpectraMax M5, Molecular Devices, Sunnyvale, CA, USA) was utilized to measure the absorbance of the collected BPW solutions at an OD of 340. Standard curves were prepared using a lactic acid standard (Sigma-Aldrich).

### Determination of relative gene transcription levels

Quantitative reverse transcription PCR (qRT-PCR) was used to determine the effects of the compounds on the expression of mRNAs *smu.238, gtfB, gtfC, gtfD, gbpA, gbpC, smu.44, smu.46, ciaH, ldH*, and *aguA*. The *S. mutans* strains were cultured overnight. The isolation, purification, and reverse transcription of total bacterial RNA into cDNA was performed as described previously (Zuo et al., 2017). qRT-PCR was performed using the Bio-Rad CFX96 system (Bio-Rad, Marnes-la-Coquette, France). All primers used are listed in Table 2. The reaction mixture (20 μL) contained 1X SYBR green PCR master mix (Takara-Bio, Otsu, Japan), template cDNA, and forward and reverse primers (10 mM each). The thermal cycling conditions were as follows: initial denaturation at 95°C for 30 s followed by 40 cycles of 95°C for 15 s and 60°C for 30 s. An additional step at 95°C for 15 s and 60°C for 1 min (0.05°C s^−1^) was used to establish a melting curve.

### Statistical analysis

All experiments were performed at least in triplicate and reproduced three separate times. Statistical analysis was conducted with SPSS software version 21.0 using one-way analysis of variance and Tukey's multiple comparisons test. *P* < 0.05 was considered statistically significant.

## Results

### AMC metabolism was partially restored by SahH supplementation

*Sm*.Δ*luxS*/pIB169 and *Sm*.Δ*luxS*/*sahH* strains were successfully constructed and the transformation and expression of *sahH* gene in *Sm*.Δ*luxS*/*sahH* were verified by PCR and RT-PCR (Figure S1). A HPLC-MS/MS method was developed and the standard curves of the four metabolites were shown in Figure S2. By using the HPLC-MS/MS method, changes in the synthesis of metabolites in the four strains were investigated, and significant differences were observed among these strains. Under the culture conditions used (in the stationary phase, Figure 1B), the amount of SAM and MET was one to two orders of magnitude higher than the amount of SAH and HCY. Furthermore, the amount of SAH and SAM was significantly higher in *Sm*.Δ*luxS* and *Sm*.Δ*luxS*/pIB169 strains than in their counterparts, and HCY was higher in *Sm*.wt (Figure 1C, *P* < 0.05). The results suggested that complementation of SahH partially restored the AMC metabolism in *S.mutans*.

### Decrease in biofilm formation was restored by SahH-original metabolic complementation

**Figure 2:**
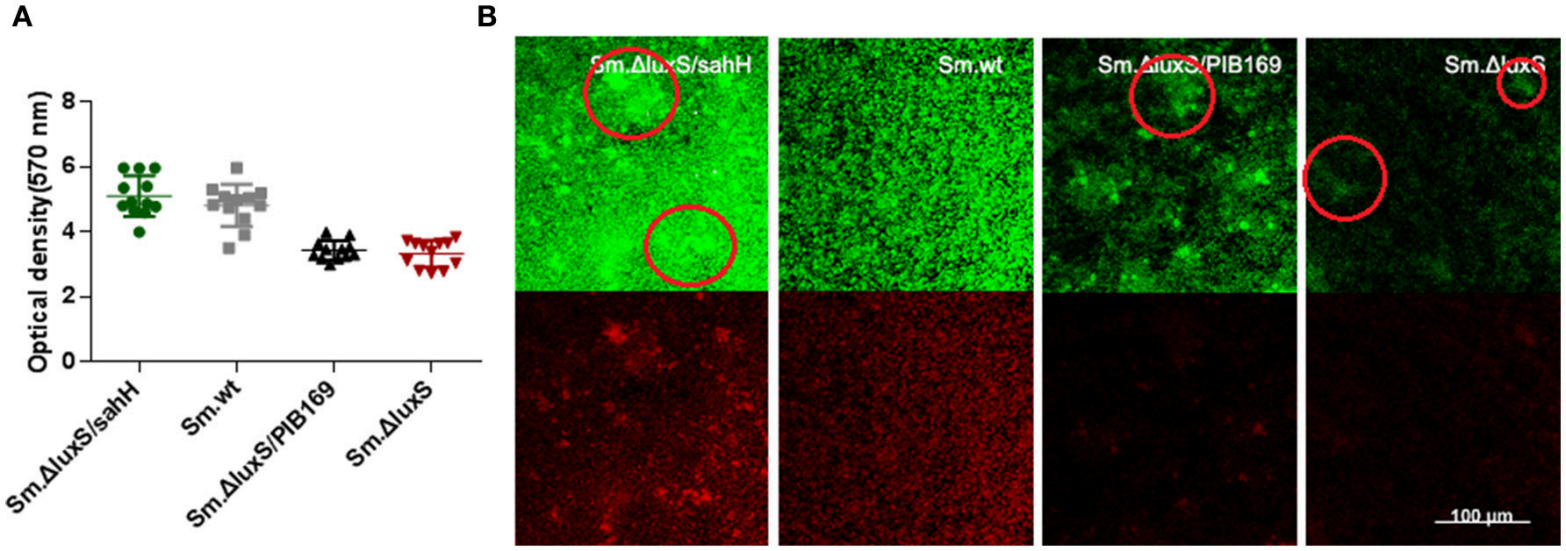
Quantification of biofilms by CV staining and CLSM of biofilms formed by *S. mutans* and derivative strains. **(A)** Quantification of biofilms. Two independent experiments were performed (*n* = 3 for each strain). The results are shown as mean ± *SD*, and the asterisks represent a significant difference (*P* < 0.05) compared to the *Sm*.Δ*luxS* strain. **(B)** The CLSM of biofilms. Double-labeling imaging of bacteria (green) and EPS (red) of biofilm formed on the surface of glass coverslips, showing the topographic features of biofilm. Images are representative of compressed biofilms in strains *Sm*.wt, *Sm*.Δ*luxS, Sm*.Δ*luxS*/*sahH, Sm*.Δ*luxS*/pIB169. The distinct bulges which were resulting from high-density cell aggregates in *Sm*.Δ*luxS*/*sahH, Sm*.Δ*luxS*, and *Sm*.Δ*luxS*/pIB169 were circled. Images were randomly captured from each sample.

The biofilm formation of *S. mutans* plays an essential role in the etiology and pathogenesis of dental caries. In our study, quantification of biofilm was conducted to investigate whether SahH complementation could restore the *luxS*-deletion induced alteration of biofilm formation. The amount of biofilm was quantified by staining with 0.1% CV and absorbance reading at an OD of 570 nm. *luxS* deletion in *Sm*.Δ*luxS* caused a decrease in biofilm mass compared to *Sm*.wt (Figure 2A). The decrease was completely restored by the supplementation of transgenic SahH, and the amount of biofilm was similar between *Sm*.Δ*luxS*/*sahH* and *Sm*.wt. In contrast, the amount of biofilm was not restored in *Sm*.Δ*luxS*/pIB169 and was decreased compared to *Sm*.wt. Thus, a contributory effect of the functional AMC on biofilm formation was verified by quantification.

### Impairment of biofilm formation was partially restored by transgenic SahH

To further investigate the restoration of biofilm formation by SahH, a structural analysis was made with CLSM. Compared with the dense, uniform biofilm with complete coverage of the slide surface by *Sm*.wt, the biofilm formed by *Sm*.Δ*luxS* was heterogeneous and organized, cell aggregates, and conspicuous empty surfaces were scattered throughout the matrix (the cell aggregates were circled in Figure 2B). Besides, no textural repair was observed in *Sm*.Δ*luxS*/pIB169 biofilms. However, for the *Sm*.Δ*luxS*/*sahH* biofilm, although pronounced bulges resulted from the aggregation of high density cells could still be found, a dense structure was restored and a smaller empty surface was present. This suggested that partial restoration of biofilm texture was obtained by *Sm*.Δ*luxS*/*sahH* due to complementation of SahH (Figure 2B).

### Acid tolerance defect was restored by SahH supplementation

**Figure 3:**
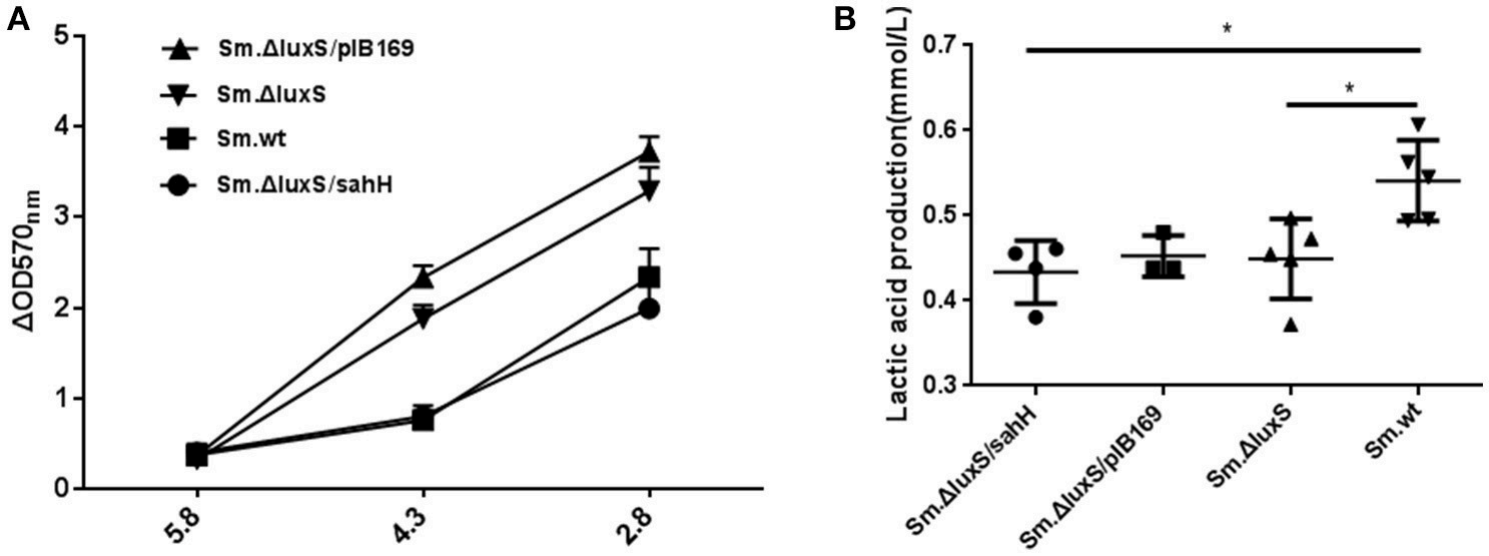
Acid tolerance and lactic acid production of four bacterial strains in the presence of biofilms. **(A)** Acid tolerance of four bacterial strains. ΔOD570 nm is the difference in optical density at 570 nm between the experimental groups (pH 4.3 or pH 2.8) and reflects the acid-inhibition effect. The results are shown as mean ± SEM. **(B)** Variability in acid production in biofilms from the four strains. Lactic acid production was higher in the *Sm*.wt strain than in *Sm*.Δ*luxS* and *Sm*.Δ*luxS*/*sahH* strains. ^*^Significant differences at *P* < 0.05.

For phenotypic confirmation of the transcriptional profiling, acid tolerance of the four *S. mutans* strains was evaluated by exposure to low pH (5.8, 4.3, and 2.8), and to neutral medium (pH 7.4) as a control. Decreased biofilm production at lower pH was a reflection of acid inhibition and thus reversed acid tolerance. The results indicated that a weakly acidic environment (pH 5.8) did not strongly suppress biofilm formation. However, at pH 4.3, biofilm formation was decreased, and this decrease was categorized into two groups: a higher-decrease group, including *Sm*.Δ*luxS* and *Sm*.Δ*luxS*/pIB169; and a lower-decrease group, including *Sm*.wt and *Sm*.Δ*luxS*/*sahH* (*P* < 0.05). This suggested better resistance to acid inhibition by *Sm*.wt and *Sm*.Δ*luxS*/*sahH* than *Sm*.Δ*luxS* and *Sm*.Δ*luxS*/pIB169. A similar profile was observed at pH 2.8 (Figure 3A). Hence, the greater sensitivity of *S. mutans luxS* null strain to acid inhibition that resulted from luxS deletion was compensated by SahH complementation.

### Production of lactic acid and extracellular polysaccharides

*S. mutans* in biofilms can metabolize carbohydrates to acids (mainly lactic acid), causing demineralization of the tooth structure and EPS are extremely important in the processes of biofilm formation and stabilization. Therefore, the discoveries of lactic acid production and EPS synthesis abilities have great significance. In the study, the ability of the four bacterial strains to produce lactic acid and extracellular polysaccharides were investigated using the lactate dehydrogenase and anthrone-sulfuric method. The results indicated that lactic acid production in *Sm*.wt was higher than that in *Sm*.Δ*luxS* and *Sm*.Δ*luxS*/*sahH* when cultured for 24 h (Figure 3B, *P* < 0.05). However, there was no significant difference between *Sm*.Δ*luxS*/*sahH* and *Sm*.Δ*luxS*. Thus, the lactic acid production ability was not restored by SahH supplementation. Besides, the anthrone-sulfuric results indicated that there was no significant difference in EPS (water-soluble and insoluble) between *Sm*.Δ*luxS, Sm*.wt, and *Sm*.Δ*luxS*/*sahH* (Figure S3, *P* > 0.05). The EPS of biofilm formed by *S. mutans* and derivatives were showed by CLSM. The results (Figure 2B, stained red) showed that with the changes in the number of bacteria in the biofilm, the amount of bacterial EPS also changes.

### Transcriptional alterations of target genes were partially restored by supplementation of transgenic SahH

**Figure 4:**
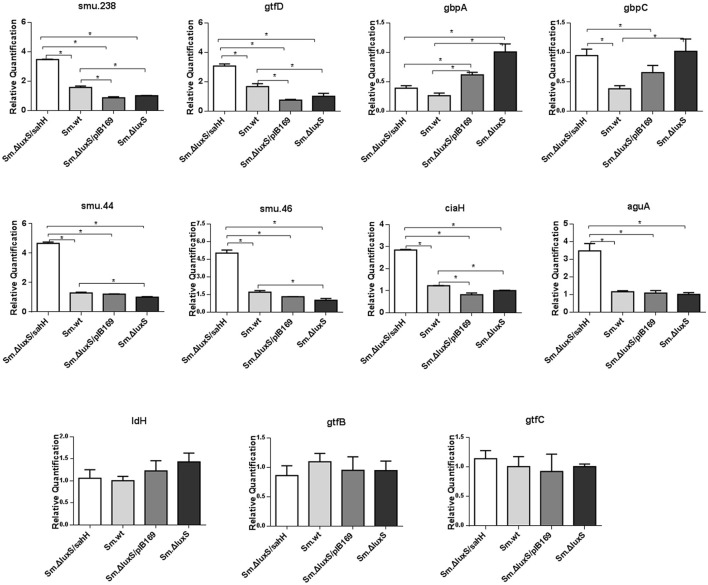
Transcriptional comparison of studied genes with qRT-PCR. The 11 target genes were relevant to biofilm formation (*smu.238, gtfD, gbpA*, and *gbpC*), aciduricity (*smu.44, smu.46, ciaH*, and *aguA*), acid generation (*ldH*), and synthesis of extracellular polymeric substances (*gtfB, gtfC*). RNAs were quantified with qRT-PCR and normalized to 16S rRNA transcripts. Results represent mean ± *SD* of relative quantification. ^*^Significant differences at *P* < 0.05.

The transcriptional changes of 11 genes involved in biofilm formation, acid tolerance, lactic acid production, and EPS synthesis in *S. mutans* were quantified by qRT-PCR. Four genes involved in biofilm formation (*smu.238, gtfD, gbpA*, and *gbpC*), four genes related to acid tolerance (*smu.44, smu.46, ciaH*, and *aguA*), two genes involved in extracellular polysaccharide synthesis (*gtfB* and *gtfC*), and one gene related to lactic acid production in *S. mutans* (*ldH*) (Yamada and Carlsson, 1975; Koo et al., 2006; Sztajer et al., 2008; Wen et al., 2011) were chosen to evaluate gene transcription. Six genes (*smu.44, smu.46, ciaH, smu.238, gtfD*, and *gbpA*) were restored by SahH supplementation, and the most significant changes were observed in smu.46, with a maximal downregulation of 69% by *luxS* mutation and a maximal upregulation of 196% by transgenic SahH (Figure 4). Although *luxS* mutation did not induce transcriptional changes in the *aguA* gene (*P* > 0.05 between *Sm*.Δ*luxS* and *Sm*.wt), SahH supplementation caused a strong upregulation, with an increase of 200% relative to *Sm*.wt. However, the upregulation of *gbpC* gene by *luxS* mutation was not significantly restored by *sahH* supplementation, and the expression of *gtfB, gtfC*, and *ldH* was not compensated in *Sm*.Δ*luxS*/*sahH*.

## Discussion

AI-2-dependent QS is considered a primary contributor to the impairment of biofilm formation and acid tolerance by *luxS*-deletion in *S. mutans* (Li et al., 2002; Merritt et al., 2003). However, evidence has suggested that many changes induced by *luxS*-mutation failed to respond to exogenous AI-2 (Sztajer et al., 2008). For this reason, the predominant role of QS in LuxS regulation was questioned. Moreover, *luxS* deletion caused AI-2-mediated QS disruption and metabolic disorders. Therefore, the latter could be a contributor to the disruptions caused by *luxS* mutation. In our study, the AMC defect in the *luxS* mutant strain with an AI-2 defect phenotype was restored using the transgenic SahH heterologously expressed in *Sm*.Δ*luxS*, and the *sahH* mRNA was exclusively expressed in *Sm*.Δ*luxS*/*sahH* (data not shown). The difference in the amount of AMC metabolites for four important cariogenic phenotypic effects (biofilm formation, acid tolerance, lactic acid production, EPS synthesis ability) and the relative gene transcription levels of the four strains were confirmed.

Changes in metabolite levels by *luxS* mutation and *sahH* supplementation were determined to elucidate the AMC in *S. mutans*. The AMC metabolites were extracted, identified, and quantified with HPLC-MS/MS. The results revealed that the concentration of SAH, SAM, and HCY were significantly different between the *S. mutans* strains. After culturing these strains overnight, the levels of SAM and SAH were significantly higher in *Sm*.Δ*luxS*/pIB169 and *Sm*.Δ*luxS* than in *Sm*.Δ*luxS*/*sahH* and *Sm*.wt. It can be speculated that, because of the SahH pathway which presumably converts SAH to HCY, the levels of SAM and SAH in the *Sm*.Δ*luxS*/*sahH* strain were lower than those in *Sm*.Δ*luxS*/pIB169 and *Sm*.Δ*luxS* but similar to those in *Sm*.wt, remained at a relatively lower level. In contrast, SAM and SAH were unable to normal metabolize in ΔluxS/plasmid and *Sm*.Δ*luxS* because mutations in *luxS* genes inhibited the LuxS enzyme. Consequently, the concentrations of SAH and SAM continued to increase in *Sm*.Δ*luxS*/pIB169 and *Sm*.Δ*luxS*. Therefore, the metabolism of SAH and SAM was complemented by SahH supplementation.

HCY was significantly lower in the *Sm*.Δ*luxS, Sm*.Δ*luxS*/pIB169 compared with *Sm*.wt. This difference may be because of the lack of recycling of interactive metabolites in these mutant strains and other changes in the SahH pathway in *Sm*.Δ*luxS*/*sahH* strain. HCY can be synthesized *de novo* from cystathionine using sulfate and oxaloacetate as precursors in a series of biochemical steps, and this synthesis served to replenish the AMC metabolites (Walters et al., 2006). Halliday et al. (2010) considered that the *de novo* synthesis mechanism explained why HCY was still detectable in LuxS-mutant strains. The significantly lower concentration of HCY in AMC mutants suggested that under the growth conditions used, the loss of the pool concentration of HCY was not being compensated for by increased *de novo* synthesis. Instead, the cycle was likely restored via the uptake of MET from the growth medium. The concentration of MET in bacterial cells was similar between the four evaluated strains. MET is a crucial amino acid, it has extensive cellular functions in addition to its role in the supply of activated methyl groups (Sekowska et al., 2000). The similar concentration of MET in bacterial cells between the four evaluated strains may reflect the need for the bacteria to maintain a metabolite pool with a relatively high and stable concentration of the metabolite. Thus, it can be speculated that part of the metabolic defect caused by mutations in *luxS* genes in *S. mutans* was compensated by the supplementation of SahH.

In this study, the biofilm mass was markedly decreased by *luxS* deletion, which agrees with the results of other studies (Merritt et al., 2003; Yoshida et al., 2005). It is of note that when the metabolic disruption was reversed by SahH supplementation, the decrease in biofilm mass in *Sm*.Δ*luxS*/*sahH* was reversed to a level similar to that of *Sm*.wt. Biofilm quantification supported the predominant role of methionine metabolism in biofilm formation in *S. mutans*. In addition, along with the changes described previously, the expression levels of genes *smu.238, gtfD* and *gbpA* were restored in *Sm*.Δ*luxS*/*sahH*. The *gbpC* gene was upregulated by *luxS*-deletion. GbpC is believed to promote dextran-dependent bacterial aggregation and biofilm formation (Sato et al., 2002) and was not restored by SahH supplementation. However, other unknown mechanisms may be involved in the transcriptional regulation of *gbpC* by *luxS*.

The biofilm structure of the four bacterial strains was evaluated with CLSM. Our results revealed that *luxS*-mutation impaired biofilm formation, and this result agrees with those of previous studies (Wen and Burne, 2004; Lebeer et al., 2007). In contrast to the complete restoration of biofilm formation by SahH, the parental structural phenotype of homogeneous biofilm was not fully rescued in *Sm*.Δ*luxS*/*sahH*. However, although the distinct bulges resulting from cell aggregates were found in *Sm*.Δ*luxS*/*sahH* biofilm, dense structure was regained and smaller fissures were present. Therefore, the methionine metabolism may partially contribute to the structure phenotype of biofilm.

Acid tolerance, acid production, and extracellular polysaccharides are crucial cariogenic traits of *S. mutans* (Huang et al., 2002; Wen and Burne, 2004). In this study, the aciduric assay revealed that at pH 4.3 and 2.8, *Sm*.Δ*luxS*/*sahH* and *Sm*.wt had a higher ability of acid resistance than *Sm*.Δ*luxS* and *Sm*.Δ*luxS*/pIB169, and the increased ability to restore genes *smu.44, smu.46*, and *ciaH*. This result indicates that the acid tolerance defect caused by *luxS* mutation in *Sm*.Δ*luxS* was repaired by transgenic SahH supplementation. The *aguA* gene encodes an enzyme that produces ammonia, and the enzyme can reduce the intracellular pH in streptococci that are less acid-tolerant (Griswold et al., 2006). Sztajer found that *aguA* transcription was strongly downregulated (72.97-fold) by *luxS* mutation (Sztajer et al., 2008). In contrast, the transcriptional level of *aguA* was non-significantly downregulated by *luxS* mutation in our study. However, transcription was markedly increased by SahH supplementation, which supports a potential regulatory effect of the functional AMC on gene expression.

Few studies to date have evaluated the variability in acid generation by knocking off the *luxS* gene in *S. mutans*. In our study, the ability of biofilms to produce lactic acid in *Sm*.wt was higher than that of the other three strains when cultured 24 h. Furthermore, no significant changes in the expression of the *ldH* gene were observed between the four strains. For this reason, acid production may not be responding to SahH supplementation. In this respect, a defect in LuxS may lead to a decline in the production of lactate dehydrogenase. With regard to the production of exopolysaccharides, Ye et al. (2008) found an increase in EPS production in *Vibrio alginolyticus*. However, the amount of EPS in *Sm*.wt, *Sm*.Δ*luxS*, and *Sm*.Δ*luxS*/*sahH* was similar in this study. We hypothesize that AI-2-mediated QS impairment or methionine methyl metabolism defect does not affect EPS synthesis in *S. mutans*, and the transcriptional levels of *gtfB* and *gtfC* confirmed this hypothesis.

However, apart from AMC metabolism, many intracellular activities are found to be involved in LuxS-related functions, including the AI-2-LsrK axis in *Yersinia pestis* (Fitts et al., 2016) and the LuxS/AI-2/Rbf regulatory cascade in *Staphylococcus aureus* (Ma et al., 2017). The potential AI-2 receptors may also have a role in the LuxS system, including LuxP (in *V. harveyi*) (Chen et al., 2002), LsrB (in *Bacillus cereus, Escherichia coli*, and *S. enterica*), and RbsB (in *Haemophilus influenza*) (Pereira et al., 2013; Ali et al., 2017). Moreover, environmental stress conditions may affect the LuxS/AI-2 system (Park et al., 2017). The relationship between these intracellular and extracellular processes and methyl metabolism is unclear and more studies are needed to elucidate these mechanisms.

## Conclusions

Our results demonstrated the occurrence of changes in AMC metabolites, biofilm formation, acid tolerance, lactic acid production, EPS synthesis ability, and transcription levels of related genes in four *S. mutans* stains (*Sm*.Δ*luxS, Sm*.Δ*luxS*/pIB169, *Sm*.Δ*luxS*/*sahH*, and *Sm*.wt), and provided evidence of the crucial contribution of methyl metabolism to LuxS-regulated functions. In conclusion, the study verified the hypothesis that apart from quorum-sensing, methionine methyl metabolism also contributes to LuxS regulation in *S. mutans* to a significant degree. However, the growth and other factors, like metabolic differences, were not normalized in the cultivation of biofilm in the study, which may have a certain impact on the CV staining results. Besides, exogenous transformation of *sahH* gene has not completely restored the *luxs*-mutant strain's function defects, like the concentration of HCY and lactic acid production ability. Thus, further studies are needed to elucidate the mechanisms underlying the change caused by *luxS* mutations.

## Author contributions

ZH and RM: conceived and designed the work; XH and YW: performed all the experiments, analyzed the results; CN, LG, XH and WJ: contributed reagents, materials, analysis tools; LG, WL, XH, KY and WJ: wrote the paper, ZH and RM: revised the manuscript.

### Conflict of interest statement

The authors declare that the research was conducted in the absence of any commercial or financial relationships that could be construed as a potential conflict of interest.

## Abbreviations

AMC: Activated methyl cycle
SAM: S-adenosylmethionine
SAH: S-adenosylhomocysteine
HCY: Homocysteine
MET: Methionine
[^13^CD_3_]MET: [^13^CD_3_] methionine
*Sm*.wt: *S. mutans* UA159
*Sm*.Δ*luxS*: *S. mutans luxS*-null strain
*Sm*.Δ*luxS/sahH*: *S. mutans luxS*-null strain with pIB-sahH
*Sm*.Δ*luxS*/pIB169: *S. mutans luxS*-null strain with pIB169.
