# Supplementary figures and images for: The Impairment of Methyl Metabolism From luxS Mutation of Streptococcus mutans
Source: Front Microbiol. 2018 Mar 12;9:404. doi: 10.3389/fmicb.2018.00404 (PMC5890193; doi:10.3389/fmicb.2018.00404)

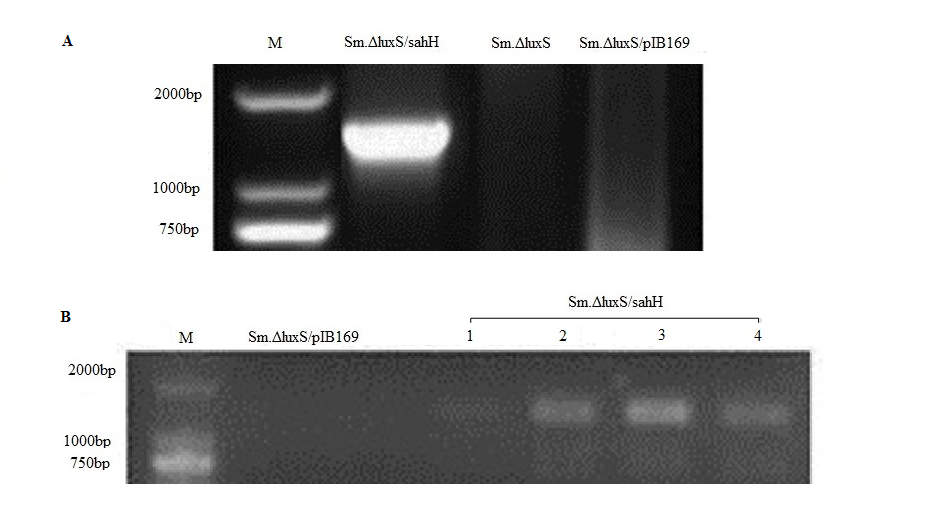

Supplement: Supplementary file 1 [file Image1.TIF]

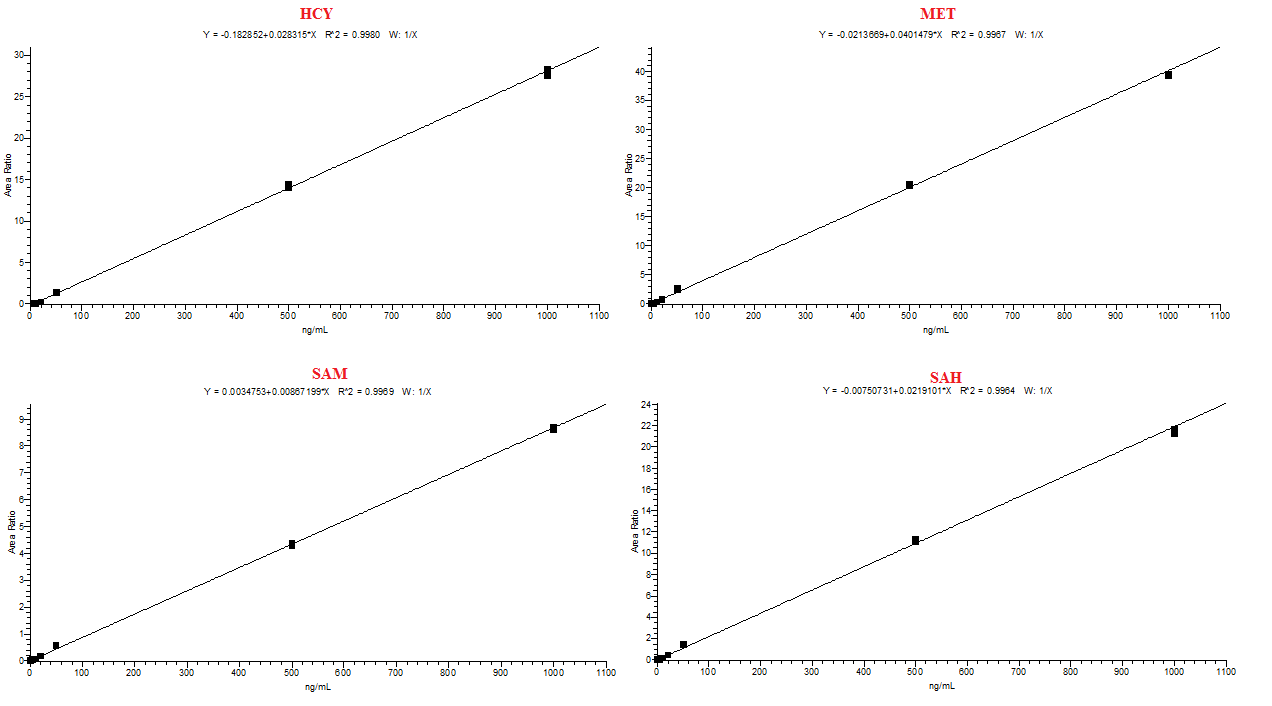

Supplement: Supplementary file 2 [file Image2.TIF]

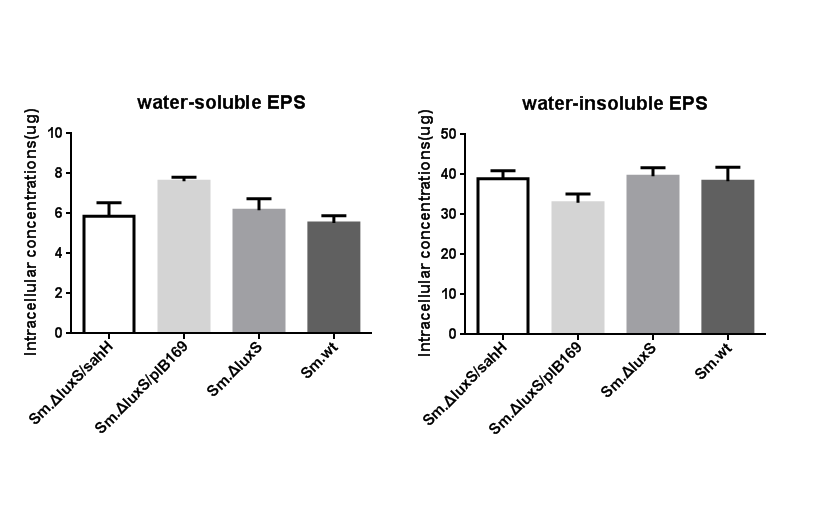

Supplement: Supplementary file 3 [file Image3.TIF]
