# Supplementary material for: The Impairment of Methyl Metabolism From luxS Mutation of Streptococcus mutans
Source: Front Microbiol. 2018 Mar 12;9:404. doi: 10.3389/fmicb.2018.00404 (PMC5890193; doi:10.3389/fmicb.2018.00404)
Supplement: Supplementary file 4 [file Presentation1.PDF]

The impairment of methyl metabolism from luxS mutation of Streptococcus mutans

Running title: Metabolism Contributes to luxS-mutation Impairments

Xuchen Hu[1#], Yuxia Wang[2#], Li Gao[1], Wenxin Jiang[1], Wenzhen Lin[1], Chenguang Niu[1], Keyong Yuan[1], Rui Ma1*, Zhengwei Huang1*.

Supplementary Information

The supplementary information includes:

Figure S1. The *sahH* gene in Sm.ΔluxS/sahH strain was confirmed.

Figure S2. The standard curves of standard solutions (HPLC-MS/MS)

Figure S3. Synthesis of extracellular polysaccharides in four strains.

Supplementary Materials:

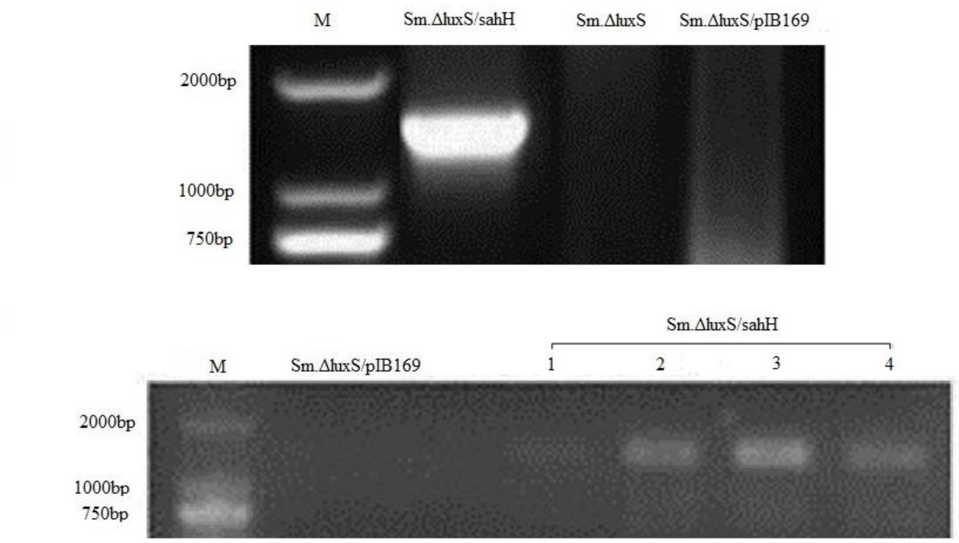

Figure S1. The *sahH* gene in Sm.ΔluxS/sahH strain was confirmed. (A) PCR result showed that sahH gene (about 1400bp) was successfully transformed into Sm.ΔluxS/sahH strain. (B)The mRNA of sahH gene was expressed in Sm.ΔluxS/sahH strain by RT-PCR. Four repetitions were set and the group with the most active mRNA transcription was selected for subcultures.

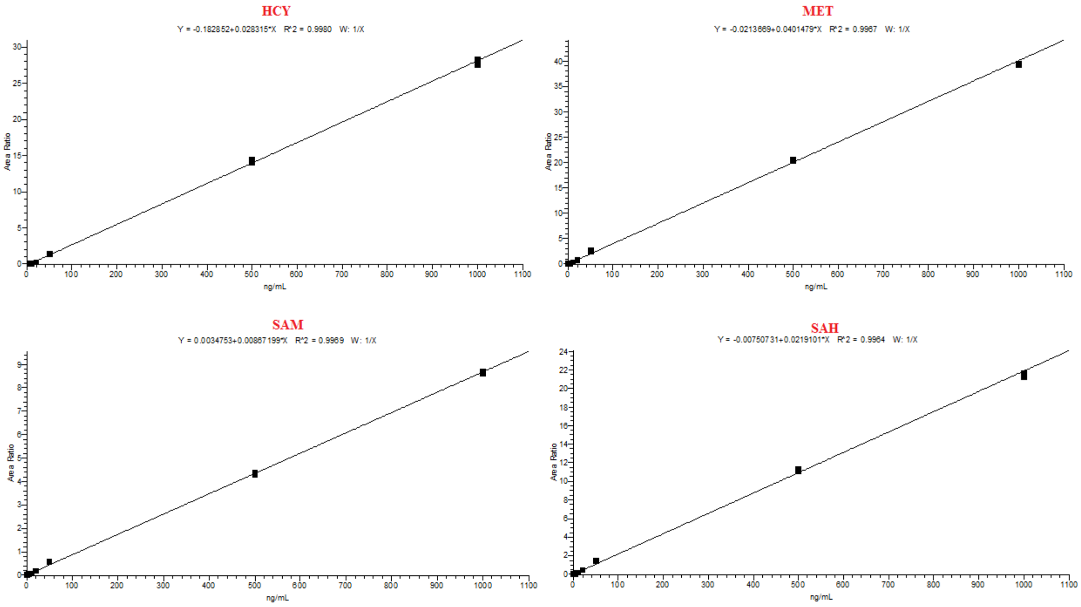

Figure S2.The standard curves of the four metabolites which were determined by HPLC-MS/MS.

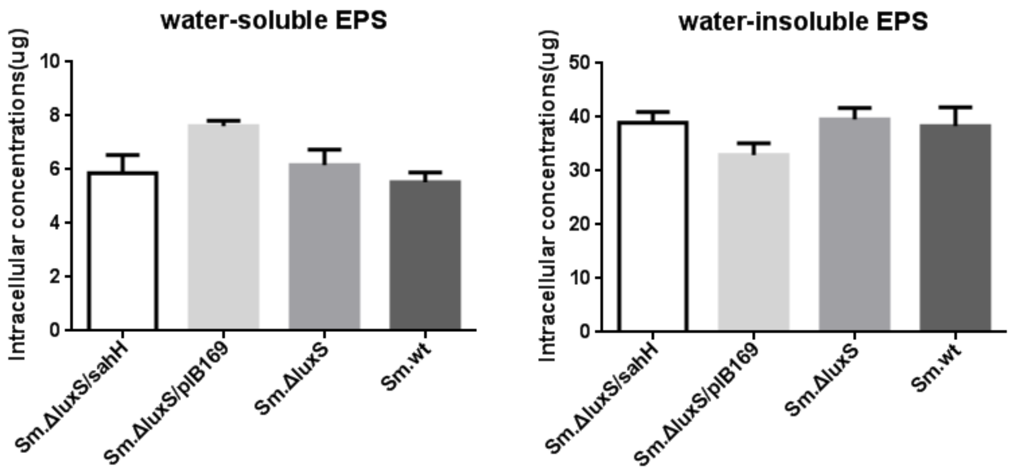

Figure S3. Synthesis of extracellular polysaccharides in Sm.wt, Sm.ΔluxS, Sm.ΔluxS/sahH, and Sm.ΔluxS/pIB169 strains. The quantitative data on the amount of water-solute and water-insoluble polysaccharides in *S. mutans* strains were determined with the anthrone-sulfuric method. The levels in Sm.ΔluxS, Sm.wt, and Sm.ΔluxS/sahH were not significantly different (*P*>0.05).
